# The causal relationship between circulating cytokines and critically ill COVID-19: A bidirectional Mendelian randomization analysis

**DOI:** 10.7189/jogh.12.05010

**Published:** 2022-02-19

**Authors:** Yu Yang, Yang Xiaohui, Sui Miao, Zhong Yingshuo

**Affiliations:** Endocrinology Department, Affiliated Zhongshan Hospital of Dalian University, Dalian, Liaoning, China

## Abstract

**Background:**

In this study, we performed a bidirectional mendelian randomization analysis on circulating cytokines and critically ill COVID-19.

**Methods:**

Both the exposure and outcome data were obtained from public genome wide association study (GWAS) database. We extracted independent instrumental variables from exposure at genome level significance (*P* < 5 × 10^−8^). Wald ratio or inverse variance weighted (IVW) method were used for estimating the causal relationships between circulating cytokines and critically ill COVID-19.

**Results:**

Only IL5 (cytokines to critically ill COVID-19 direction) and bNGF, IL8 (critically ill COVID-19 to cytokines direction) showed suggestive causal relations. However, these associations lost significance after FDR correction. Another validation data set of critically ill COVID-19 did not confirm these associations, either.

**Conclusions:**

Our Mendelian randomization did not find causal relationships between analyzable circulating cytokines and critically ill COVID-19.

The newly emerging COVID-19 (Corona virus disease 2019) is continuing to threaten the public health system throughout the world. The clinical manifestation of COVID-19 is complex, and substantial variations have been observed in the severity of disease. Most infected persons have mild or even no symptoms. However, the critically ill COVID-19 patients with severe respiratory failure and acute respiratory distress syndrome (ARDS) might need intensive therapy and have higher mortality rates [1].

Up to now, the exact pathogenesis of critically ill COVID-19 and its associated respiratory failure is poorly understood. Those individuals who are susceptible to severe infection and the immune mediated disease are strongly heritable and associated with specific genetic variants [2,3]. Several genome-wide association studies (GWAS) in critically ill COVID-19 demonstrated that, compared to the mild and moderate subtype, the severe COVID-19 might have a distinct pathophysiology [4,5]. COVID-19 infection is usually accompanied with an adverse host event “cytokine storm”, which is characterized by an aggressive inflammatory response with the release of a large amount of pro-inflammatory cytokines [6-8]. Several studies have suggested that, the cytokine storm correlated directly with lung injury, multi-organ failure, and unfavorable prognosis of critically ill COVID-19 [8]. Moreover, effective strategies targeting cytokines during the treatment of severe COVID-19 patients could improve the survival rates and reduce mortality [9,10].

However, whether these cytokines have causal relationship with critically ill COVID-19 remains to be elucidated. The mendelian randomization (MR) approach, which uses genetic variants as instrumental variables, has been adopted as a powerful method to evaluate the causal relationship of potential exposure on certain disease [11]. The genetic variants, usually SNPs, are randomly assigned during gamete formation and less likely to be affected by confounding. Therefore, this kind of MR study is analogous to a randomized controlled trial, and can attain stable and convincing causal effects between exposures and outcomes [12,13].

A better understanding of the role of inflammatory biomarkers in COVID-19 is needed, which may help prevent the incidence and aid in the development of novel therapeutic targets. In this study, we performed a bidirectional MR analysis to assess the causal direction between cytokines with critically ill COVID-19 using GWAS summary data. The aim of the present study was to (1) estimate the causal relationships between cytokines and the development of critically ill COVID-19; (2) appraise the evidence for direction and robustness in the estimated etiological associations; (3) investigate potential genetic variants that lead to the variation of COVID-19 related cytokines, which might also contribute to the progress of critically ill COVID-19.

## METHODS

### Study design

In this study, we conducted a bidirectional MR test to decipher the causal effect of circulating cytokines with critically ill COVID-19 using publicly available genetic association summary data (two-sample MR). The underlying idea of the MR method is to use genetic variants as instrumental variables (IVs) to link a risk factor (‘exposure’) to a health trait (‘outcome’). In order to represent a valid IV, a genetic variant need to fulfill three assumptions: 1) is associated with the exposure, 2) only affect an outcome via the exposure, 3) and is independent of confounders. In this MR study, exposures and outcomes in the cytokines to critically ill COVID-19 direction are circulating cytokines levels and COVID-19, respectively. For the critically ill COVID-19 to cytokines direction, the assignment is reversed.

Contributing studies have received ethical approval from their respective institutional review boards. This MR study is a secondary research from public available data. Therefore, no extra ethical approval and informed consent are required.

### Genetically determined levels of circulating cytokines

The circulating cytokines summary data were obtained from Ahola-Olli’s study [14]. In this study, the authors identified 27 genome-widely significant loci for 41 cytokines. For each cytokine, SNPs associated with circulating levels at a significance threshold (*P* < 5x10^−8^) were chosen as IVs. We performed linkage disequilibrium clumping for the significant SNPs through the European 1000 Genomes Project reference panel, and chose the independent genetic variants for further MR analysis. Overall, 24 cytokines with at least one genetic variable were identified as exposures for this study. Full GWAS summary statistics for the circulating cytokines are publicly available through the GWAS Catalog server at https://www.ebi.ac.uk/gwas/home.

### Covid-19 host genetics initiative (COVID-19 HG)

The critically ill COVID-19 summary data were obtained from COVID-19 HG GWAS meta-analysis (https://www.COVID-19hg.org/) [15]. In brief, the COVID-19 HG is an ongoing, international initiative that aims to facilitate COVID-19 host genetic study. After collecting pre-specified individual level or summary results from GWASs, it combines these data in an inverse variance weighted meta-analysis using variants with a minor allele frequency (MAF)>0.001 and imputation quality (r^2^) > 0.6. In this study, we obtained the summary statistics from data R5A2 (European population), which collects critically ill COVID-19 (n = 4792) and population controls (n = 1 054 664). For privacy issues, the summary data used in this study did not include the 23andme and UKBB cohorts.

### Statistical analysis

The MR analysis and sensitivity analysis were conducted using R platform via R package TwoSampleMR [16]. We used the standard Wald ratio estimator (when only 1 IV exists) or inverse variance weighted (IVW) method (when several IVs are available) as the primary MR analysis for estimating the causal relationships between circulating cytokines and critically ill COVID-19 [17]. FDR (false discovery rate) correction was applied to correct for multiple comparisons, and the *P* values after FDR correcttion were described as *P*.adjust. A *P* < 0.05 before FDR correction was considered as suggestive for association.

The statistical power calculations for the MR analysis were performed through online tool at https://sb452.shinyapps.io/power/ [18]. Briefly, for the cytokines to critically ill COVID-19 direction, the outcome was binary. We computed the power to detect 1.2 causal effects per SD change in exposure for each MR analysis at a 0.05 significance level. While for the other direction, the outcomes were continuous. Causal effect per SD change of 0.2 at a 0.05 significance level was adopted for power estimation.

In order to assess the robustness of the MR results and potential violations of the assumptions underlying MR, we performed several kinds of sensitivity analyses. These sensitivity analyses mainly include heterogeneity tests, horizontal pleiotropy and leave-one-out analysis.

Heterogeneity test, which assess the compatibility of IVs in causal effects, is an important indicator of potential violation of IV assumptions [19]. We conducted Cochran's Q test and *I^2^* statistic to compute the heterogeneity of causal estimates from each genetic variant. The MR-Egger regression was performed to assess potential directional pleiotropy of genetic variants. The slope of the MR-Egger regression can give pleiotropy corrected causal estimates, and the magnitude of the intercept can provide an estimate of the degree of pleiotropy. If the intercept differs from zero, there is evidence of directional pleiotropy. In addition, the MR-PRESSO method was applied to detect and correct for the horizontal pleiotropic outlier. Briefly, it regresses the IV-outcome and IV-exposure association to conduct a global test of heterogeneity. Then, it compares the observed distance of each SNP from the regression with the distance expected under the null hypothesis of no pleiotropy. Finally, the leave-one-out analysis is performed to evaluate whether the MR estimate is biased by any single SNP that might have a substantial horizontal pleiotropic effect. In this method, we can re-estimate the effect by sequentially dropping one SNP at a time. If the dropped SNP lead to a dramatic change in the estimate, it may have horizontal pleiotropic effect.

## RESULTS

### Instrument variables in this study

In order to eliminate potential linkage disequilibrium (LD) of genetic variants, we performed strict clumping with an R^2^ threshold of 0.01 after extracting genome-wide associated SNPs (*P* < 5 × 10^−8^) from exposure GWAS data. Because the exposure and outcome population are both from European population, European samples in the 1000 genomes project reference data set is chosen as the reference panel for clumping.

In the cytokines to critically ill COVID-19 direction, 80 independent SNPs remained and were utilized as instruments for 24 cytokines following clumping (**Table 1**). Other 17 cytokines were not suitable for exposure due to lack of appropriate IVs. Among the 24 cytokines for exposure, 8 cytokines have only 1 associated SNP. While the other 16 cytokines were associated with multiple IVs, and MIP1b having the most 18 IVs. For the critically ill COVID-19 to cytokines direction, all the 41 cytokines were eligible for outcomes. After pruning strict linkage disequilibrium clumping and data harmonization, 8 independent variants remained for further MR analysis (**Table 2**, Table S1 in the **Online Supplementary Document**). Then, we calculated F statistics for all the instrument SNPs in our analysis to avoid the weak instrument bias [20]. As shown in **Table 1** and **Table 2**, the F-statistics were well above the usual threshold of 10.

**Table 1:** MR analysis results of 24 cytokines to critically ill COVID-19 direction*

Cytokines	Number of SNPs	Variance explained	F statistics	Beta	SE	*P* value	*P*adjust
CTACK	2	0.049	184.615	0.055	0.086	0.526	0.789
bNGF	1	0.011	39.017	0.016	0.229	0.946	0.946
VEGF	7	0.045	320.340	-0.094	0.057	0.103	0.786
TRAIL	12	0.245	2074.603	-0.071	0.044	0.106	0.786
TNFb	2	0.104	173.738	0.066	0.090	0.462	0.786
SCGFb	3	0.071	266.890	0.095	0.091	0.296	0.786
SCF	2	0.010	87.294	-0.443	0.311	0.155	0.786
IL16	3	0.092	342.450	-0.005	0.063	0.936	0.946
RANTES	1	0.004	13.974	0.249	0.342	0.467	0.786
PDGFbb	6	0.070	592.388	-0.080	0.076	0.291	0.786
MIP1b	18	0.305	2630.814	0.062	0.066	0.344	0.786
MIG	1	0.010	38.195	-0.016	0.236	0.945	0.946
MCSF	1	0.016	13.724	-0.220	0.200	0.271	0.786
MCP1	2	0.024	203.860	-0.167	0.216	0.440	0.786
IL12p70	4	0.018	149.053	0.097	0.195	0.617	0.824
IP10	2	0.019	71.290	-0.286	0.234	0.221	0.786
IL18	3	0.041	150.175	0.015	0.125	0.905	0.946
IL17	1	0.005	41.923	-0.152	0.337	0.652	0.824
IL13	1	0.089	347.870	-0.099	0.089	0.265	0.786
IL10	1	0.005	37.121	-0.300	0.345	0.384	0.786
HGF	2	0.015	128.368	0.067	0.143	0.636	0.824
IL5†	1	0.011	38.656	-0.395	0.182	0.030	0.720
GROa	2	0.119	444.370	-0.004	0.054	0.934	0.946
Eotaxin	2	0.027	222.288	-0.066	0.096	0.491	0.786

SE – standard error, *P*adjust – FDR (false discovery rate) corrected P value

*Explained variance was calculated as: R^2^ = (reported GWAS effect)^2^ × 2 × minor allele frequency × (1 – minor allele frequency) / variance of trait. F statistics per variant was calculated as: (Sample size - 2) × R^2^ / (1 – R^2^). The Wald ratio estimator was used for effect estimation if one single instrument was available, and the inverse variance weighted estimation method (IVW) when more than one instrument was available.

†*P* value of IL5 showed suggestive significance at the level of 0.05.

**Table 2:** MR analysis results of critically ill COVID-19 to 41 cytokines direction

Cytokines	Number of SNPs*	Beta	SE	*P* value	*P*adjust
CTACK	8	0.042	0.062	0.494	0.856
bNGF†	7	-0.093	0.046	0.044	0.574
VEGF	7	-0.048	0.033	0.145	0.688
MIF	7	-0.023	0.047	0.628	0.888
TRAIL	8	-0.010	0.034	0.766	0.894
TNFb	7	-0.047	0.087	0.590	0.888
TNFa	8	-0.018	0.057	0.755	0.894
SDF1a	8	-0.008	0.031	0.788	0.894
SCGFb	7	-0.060	0.059	0.306	0.856
SCF	7	-0.036	0.030	0.240	0.856
IL16	8	0.014	0.045	0.766	0.894
RANTES	8	0.006	0.072	0.939	0.939
PDGFbb	7	0.015	0.030	0.628	0.888
MIP1b	8	-0.026	0.041	0.520	0.856
MIP1a	7	-0.040	0.046	0.390	0.856
MIG	7	-0.017	0.059	0.777	0.894
MCSF	7	-0.087	0.055	0.116	0.688
MCP3	8	-0.126	0.088	0.151	0.688
MCP1	7	-0.033	0.030	0.278	0.856
IL12p70	6	-0.029	0.034	0.393	0.856
IP10	6	0.038	0.069	0.575	0.888
IL18	7	0.015	0.046	0.749	0.894
IL17	8	-0.037	0.031	0.225	0.856
IL13	7	-0.047	0.046	0.311	0.856
IL10	7	-0.060	0.031	0.056	0.574
IL8‡	7	-0.091	0.046	0.049	0.574
IL6	7	-0.007	0.031	0.807	0.894
IL1ra	7	-0.016	0.046	0.730	0.894
IL1b	7	-0.058	0.036	0.113	0.688
HGF	7	-0.058	0.030	0.055	0.574
IL9	8	-0.004	0.045	0.920	0.939
IL7	7	-0.036	0.047	0.445	0.856
IL5	7	-0.073	0.047	0.121	0.688
IL4	7	-0.026	0.031	0.395	0.856
IL2ra	7	-0.036	0.057	0.522	0.856
IL2	7	-0.039	0.047	0.406	0.856
IFNg	7	0.004	0.032	0.894	0.939
GROa	7	-0.075	0.078	0.334	0.856
GCSF	8	-0.003	0.030	0.910	0.939
FGFbasic	8	-0.023	0.031	0.452	0.856
Eotaxin	7	-0.020	0.031	0.522	0.856

SNP – single nucleotide polymorphism, SE – standard error, *P*adjust – FDR (false discovery rate) corrected *P* value

*The inverse variance weighted estimation method (IVW) was used in this direction, for six to eight SNPs were available.

†*P* value of bNGF showed suggestive significance at the level of 0.05.

‡*P* value of IL8 showed suggestive significance at the level of 0.05.

### Bidirectional Mendelian randomization analysis

The primary results of our MR analysis for the circulating cytokines and critically ill COVID-19 were presented in **Table 1** and **Table 2**. For the cytokines to critically ill COVID-19 direction, the only cytokine reaching statistically significant was IL5 (*P* = 0.030, OR = 0.674; 95% confidence interval (CI) = 0.472-0.963). However, this *P* value lost significance following FDR correction for testing multiple cytokines (*P*adjust = 0.720). All the other 23 cytokines did not show confirmative association with critically ill COVID-19.

For the critically ill COVID-19 to cytokines direction, we conducted 41 times MR analysis. IVW method was applied in this direction, for eight independent IVs are available. The bNGF and IL8 cytokines showed suggestive association, with *P* value 0.044 (OR = 0.911, 95% CI = 0.832-0.998) and 0.049 (OR = 0.913, 95% CI = 0.525-0.999), respectively. However, FDR correction for these *P* values did not show significance (*P*.adjust = 0.574).

### Statistical power, heterogeneity and sensitivity tests

The statistical power of our MR analysis was shown in the Table S2 in the **Online Supplementary Document**. In the cytokines to critically ill COVID-19 direction, two independent COVID-19 data sets with similar sample size and case proportion were enrolled in our study. For each COVID-19 data set, nearly half cytokines reached the power of 80%. The other cytokines with lower power values were caused by lower explained phenotype variance. The two enrolled data sets would raise the power values in certain degree. However, we could not compute the exact power for the two data sets together, due to lack of raw GWAS data. For the suggestive cytokine IL5, the estimated power was 26.1% and 26.8%, respectively. And the actual OR value were 0.674 and 0.646 in each data set, Therefore, the power to detect IL5 association with critically ill COVID-19 would reach 81.5% and 89.5%. In the critically ill COVID-19 to cytokines direction, power values were calculated based on the 8 independent SNPs (total explained variance 0.127) and sample size of 8293 [14]. At the causal effect per SD change in exposure of 0.2 and significance level of 0.05, the power would reach 100%. We performed heterogeneity and sensitivity tests for above suggestive association MR results. For the cytokines to critically ill COVID-19 direction, only IL5 showed suggestive association with COVID-19. Because there is only one IV (rs7767396) available for IL5, no heterogeneity and horizontal pleiotropy exist. As shown in the **Table 1**, this IV explained 1.2% of the variance of IL5 levels. Therefore, we queried rs7767396 through phenoscanner database [21], which is a curated database holding publicly available results from large-scale genome-wide association studies. It is indicated that, rs7767396 is also associated with serum VEGF levels.

For the critically ill COVID-19 to cytokines direction, there are eight independent IVs in effect estimates. We conducted heterogeneity and sensitivity tests to exclude potential violation of MR assumptions (**Table 3**). Cochran's Q indicated that, there are no evidence of heterogeneity for bNGF (*P* = 0.860) and IL8 (*P* = 0.983). Moreover, MR Egger regression revealed no evidence of directional pleiotropy for critically ill COVID-19 to bNGF (intercept = -0.002, *P* = 0.962) and IL8 ((intercept = -0.005, *P* = 0.904). The “leave-one-out” analysis showed the IVW causal association estimate was not driven by a single SNP, either (Figure S1 in the **Online Supplementary Document**).

**Table 3:** Heterogeneity and pleiotropy tests for bNGF and IL8 IVs in the critically ill COVID-19 to cytokines direction

	Heterogeneity test				Pleiotropy test		
**Outcomes**	**Method**	**Q value**	***I^2^***	***P* value**	**Egger intercept**	**SE**	***P* value**
bNGF	MR-Egger	2.576	1.000	0.765	-0.002	0.043	0.962
	IVW	2.578	1.388	0.860			
IL8	MR-Egger	1.051	1.952	0.958	-0.005	0.043	0.904
	IVW	1.067	1.938	0.983			

SE – standard error

### Secondary validation study

We further performed a secondary validation study for bidirectional MR analysis based on another independent critically ill COVID-19 GWAS data set (R4A2) from COVID-19 HG (Tables S3, S4 in the **Online Supplementary Document**). This validation data set R4A2 consists of 4933 critically ill COVID-19 patients and 1 398 672 healthy controls. **Table 4** showed the results of validation study for the suggestive causal association cytokines (IL5 in cytokines to COVID-19 direction, and bNGF, IL8 in COVID-19 to cytokines direction) discovered in R5A2 data set. In the cytokines to COVID-19 direction, IL5 also showed marginal association (*P* = 0.019, OR 0.646, 95% CI 0.448-0.932). However, this association lost significance after FDR correction (*P*.adjust = 0.456). The other two cytokines (bNGF, IL8) were not associated with critically ill COVID-19. The secondary validation study did not show confirmative association for these cytokines with critically ill COVID-19 in both directions.

**Table 4:** Validation study of MR results in another COVID-19 data set

Exposures	Outcomes	Beta	SE	*P* value	OR	95% CI	*P*adjust
IL5	Critically ill COVID-19	-0.437	0.187	0.019*	0.646	0.448, 0.932	0.456
Critically ill COVID-19	bNGF	-0.088	0.050	0.080			
Critically ill COVID-19	IL8	-0.087	0.050	0.081			

MR – Mendelian randomization, OR – odds ratio, CI – confidence interval

*P value of IL5 showed suggestive significance at the level of 0.05 in the cytokine to critically ill COVID-19 direction.

## DISCUSSION

To our knowledge, this is the first attempt to assess the causal effect of the circulating cytokines levels and critically ill COVID-19 through two-sample bidirectional MR approach. Our results provide evidence that there is no confirmative causal effect between analyzable cytokines and critically ill COVID-19 in bilateral directions.

While the majority affected individuals have mild or even no symptoms, severe COVID-19 is more likely in the elderly population and those with comorbidities [1]. Cumulative studies show that, the severe COVID-19 is qualitatively different from mild or moderate disease. The exact pathogenesis underlying the critically ill COVID-19 patients remain unclear. Host genetic variations may explain part of the heterogeneity for different COVID-19 outcomes. At least two biological components, susceptibility to viral infection and propensity to develop harmful pulmonary inflammation, participate in the pathogenesis of COVID-19 mortality risk [3].

Therefore, interpreting the genetic role for critically ill COVID-19 may provide pivotal insights into its pathogenesis. The published observational studies have addressed the pivotal role of aberrant cytokines affecting inflammatory injuries of the lungs, lung blood vessels or even the general organs during critically ill COVID-19 patients. However, confounding factors (smoking, lifestyle, age, obesity and T2DM), which might bias the observed estimates, are difficult to be excluded in these observational studies. The genetic IVs for MR studies are randomly allocated during gemmate formation, and less affected by confounding factors. Therefore, the MR study has become an established method for elucidating causal relationship between the exposures and outcomes in observational epidemiology.

In the present study, we identified suggestive causal associations for IL5, bNGF and IL8 with critically ill COVID-19. However, the results from the dual directions provided no causal relationship between serum cytokines and critically COVID-19. Cochran’s Q test suggested no heterogeneity among the IVs, and MR Egger regression result did not show any bias of directional pleiotropy. Moreover, another data set also validated the causal results. Therefore, bias may not exist in our MR analysis, and none of the three main MR assumptions was violated. Compared with other common diseases, the critically ill COVID-19 GWAS had moderate sample size, which would produce moderate power in detecting causal effects. In this study, the power of critically ill COVID-19 to cytokines direction was 100%. While in the other direction, the power of about half cytokines did not reach 80%. We further performed a validation study through an independent data set, which would raise the power in certain degree.

Although we did not demonstrate the confirmative causal effect of cytokines with critically COVID-19, these results did not repudiate the role of cytokine storm during the pathogenesis of critically ill COVID-19. In fact, more and more observational studies demonstrated the aberrant cytokines profile among COVID-19 patients compared to normal control individuals [22-25]. The cytokine storm might be an important mediator during severe lung injury. One reason for these discrepancies might be that, cytokine storm is not the specific pathogenesis for critically ill COVID-19. As a kind of overwhelming systemic inflammatory response, cytokine storm also occurs in several pathologic conditions, such as viral infection, cancer, severe sepsis and multi-organ failure [26]. MR study is mainly designated to extrapolate the causal association between the exposures and outcomes. Therefore, cytokine storm might be the manifestation of aberrant inner environment during critically ill COVID-19, rather than the causal reason. Another possible mechanism for lack of causal association is that, these cytokines might form a scale-free network to participate in the pathogenesis of critically ill COVID-19. These cytokines might have minor effect alone, but exert major effects when taking together, which are similar to polygenic risk score in complex traits. This assumption requires to be validated in the future study. Moreover, earlier study has revealed that, most cytokines levels, except IL1, peaked after respiratory function nadir, indicating that cytokine expression might not be the primary cause of impaired respiratory function in patients with COVID-19 [27]. And the dynamic cytokine storms and T cell lymphopenia are associated with COVID-19 severity. Sampling and measuring cytokines at one time might not reflex the dynamic fluctuations during critically ill COVID-19.

Our results might have certain clinical and public indications. As mentioned above, cytokine storm might be a common pathway in several clinical traits. SARS-Cov-2 infection might trigger aberrant cytokine profile in susceptible individuals, especially elderly persons with multiple organ failure. Effective protection and therapy for SARS-Cov-2 infection in these susceptible persons would be vital to beneficial prognosis. Therefore, the clinical decision to COVID-19 patients should be based on their personal profile and severity. It has been reported that, an immunologic memory of cytokine storm would likely persist, even after the SARS-CoV-2 infection resolves [28]. This adverse memory would raise the possibility of viral relapse or re-infection. We recommend longitudinal follow-up of COVID-19 patients with and without cytokine storm to decipher the immunological mechanisms and biomarkers for critically ill type. Our study has several limitations. First, the critically ill COVID-19 GWAS data of this study was obtained from COVID-19 HG. Compared to other traits, the coivd-19 HG has moderate sample size in COVID-19 patients. This would produce moderate power to detect the causal effect. The cumulative GWAS for COVID-19 might provide more powerful and convincible results. Second, we could not acquire independent instrument variables for 17 cytokines, including three most important pro-inflammatory cytokines of innate immune response, IL1, TNF α, and IL6. Thus, the causal association of these cytokines and COVID-19 could not be assessed. Third, the exposure and outcome population of this study are European. Other ethic data are not available yet.

## CONCLUSIONS

In conclusion, in our bidirectional MR study, no causal effect was detected between analyzable circulating cytokines and COVID-19. And the association between critically ill COVID-19 and cytokine storm need further consideration.

## Additional material


Online Supplementary Document
