# Supplementary material for: The causal relationship between circulating cytokines and critically ill COVID-19: A bidirectional Mendelian randomization analysis
Source: J Glob Health. 2022 Feb 19;12:05010. doi: 10.7189/jogh.12.05010 (PMC8934535; doi:10.7189/jogh.12.05010)
Supplement: Online Supplementary Document [file jogh-12-05010-s001.pdf]

Figure S1. Leave-one-out sensitivity analysis for critically ill COVID-19 on cytokines bNGF and IL8.

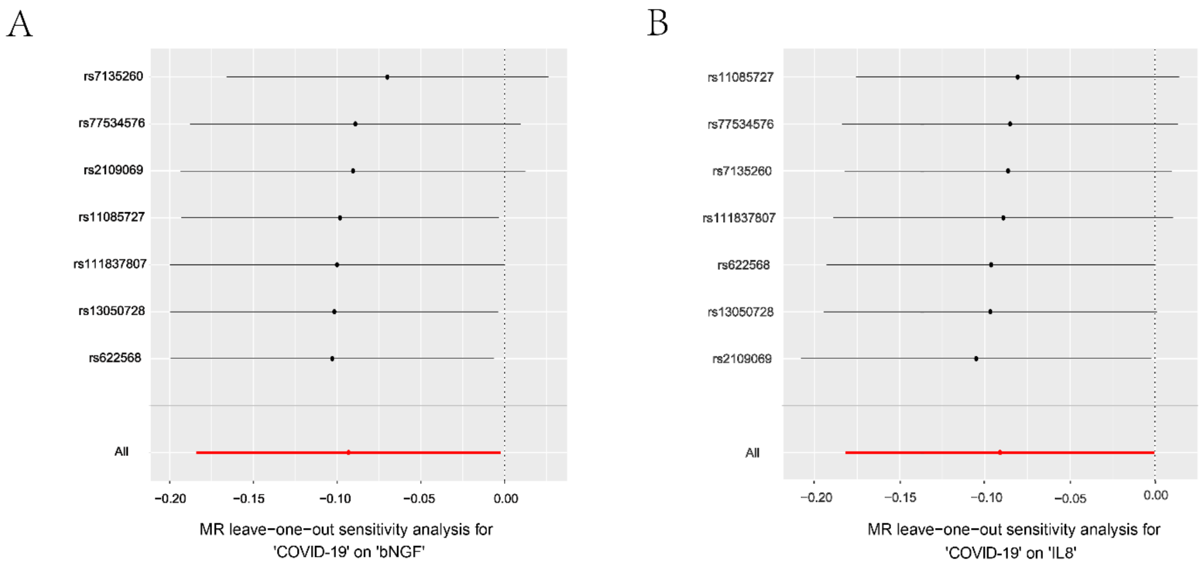

Table S1. Instrumental variables in critically ill COVID-19 to cytokines direction

SNPs	OA	EA	Beta	SE	*P* value	EAF	Variance Explained	F statistics
rs111837807	T	C	0.295	0.043	5.66E-12	0.100	0.016	256.416
rs622568	A	C	0.226	0.037	1.04E-09	0.146	0.013	173.313
rs2237698	C	T	0.237	0.040	2.41E-09	0.090	0.009	88.584
rs7135260	T	C	0.192	0.028	6.05E-12	0.674	0.016	280.517
rs77534576	C	T	0.460	0.075	8.53E-10	0.035	0.014	211.796
rs2109069	G	A	0.257	0.028	6.12E-20	0.329	0.029	894.206
rs11085727	C	T	0.173	0.029	3.74E-09	0.281	0.012	153.924
rs13050728	T	C	-0.200	0.029	2.44E-12	0.663	0.018	338.680

SE, standard error. OA, other allele. EA, effect allele. EAF, effect allele frequency.
Variance explained and F statistics were calculated as described in table 1.

Table S2. Statistical power of cytokines to critically ill COVID-19 direction in two independent datasets

**Cytokines**	**R5A2 dataset**	**R4A2 dataset**
CTACK	79.6	80.8
bNGF	26.1	26.8
VEGF	76.2	77.4
TRAIL	100	100
TNFb	98.2	98.5
SCGFb	91.9	92.6
SCF	24.2	24.8
IL16	96.9	97.2
RANTES	12.2	12.5
PDGFbb	91.5	92.2
MIP1b	100	100
MIG	24.2	24.8
MCSF	35.7	36.6
MCP1	49.6	50.8
IL12p70	39.3	40.3
IP10	41.1	42.2
IL18	72.2	73.5
IL17	14.2	14.5
IL13	96.4	96.8
IL10	14.2	14.5
HGF	33.8	34.7
IL5	26.1	26.8
GROa	99.1	99.3
Eotaxin	54.3	55.6

Table S3. MR analysis results of 24 cytokines to critically ill COVID-19 direction in validation dataset

**Cytokines**	**Beta**	**SE**	***P* value**	***P*.adjust**
CTACK	0.041	0.090	0.653	0.899
bNGF	0.185	0.241	0.442	0.899
VEGF	-0.098	0.066	0.136	0.816
TRAIL	-0.017	0.046	0.713	0.901
TNFb	0.053	0.054	0.325	0.899
SCGFb	0.012	0.092	0.894	0.937
SCF	-0.239	0.430	0.579	0.899
IL16	-0.027	0.064	0.674	0.899
RANTES	0.302	0.371	0.415	0.899
PDGFbb	-0.143	0.095	0.132	0.816
MIP1b	0.104	0.068	0.129	0.816
MIG	-0.146	0.255	0.567	0.899
MCSF	-0.189	0.209	0.364	0.899
MCP1	-0.093	0.129	0.471	0.899
IL12p70	0.021	0.167	0.898	0.937
IP10	-0.331	0.252	0.189	0.899
IL18	0.059	0.121	0.625	0.899
IL17	-0.004	0.355	0.991	0.991
IL13	-0.128	0.139	0.357	0.899
IL10	-0.212	0.361	0.557	0.899
HGF	-0.038	0.148	0.799	0.937
IL5	-0.437	0.187	0.019*	0.456
GROa	0.007	0.056	0.893	0.937
Eotaxin	-0.060	0.100	0.547	0.899

SE, standard error. OR, odd ratio. *P*.adjust. FDR corrected *P* value

* *P* value of IL5 showed suggestive significance at the level of 0.05 in the cytokines to critically ill COVID-19 direction.

Table S4. MR analysis of critically ill COVID-19 to cytokines direction in validation dataset

**Cytokines**	**Beta**	**SE**	***P* value**	***P*.adjust**
CTACK	0.028	0.067	0.677	0.777
bNGF	-0.088	0.050	0.080	0.398
VEGF	-0.049	0.035	0.168	0.574
MIF	-0.022	0.050	0.664	0.777
TRAIL	-0.022	0.038	0.559	0.721
TNFb	-0.050	0.103	0.623	0.774
TNFa	-0.045	0.059	0.445	0.721
SDF1a	-0.021	0.033	0.527	0.721
SCGFb	-0.076	0.066	0.248	0.636
SCF	-0.060	0.033	0.068	0.398
IL16	-0.005	0.049	0.920	0.920
RANTES	0.032	0.078	0.682	0.777
PDGFbb	0.019	0.033	0.555	0.721
MIP1b	-0.039	0.046	0.398	0.700
MIP1a	-0.059	0.050	0.239	0.636
MIG	-0.061	0.059	0.300	0.674
MCSF	-0.094	0.060	0.116	0.432
MCP3	-0.080	0.088	0.363	0.700
MCP1	-0.054	0.033	0.097	0.398
IL12p70	-0.039	0.033	0.234	0.636
IP10	0.006	0.061	0.917	0.920
IL18	-0.009	0.049	0.854	0.898
IL17	-0.055	0.033	0.093	0.398
IL13	-0.051	0.050	0.306	0.674
IL10	-0.086	0.034	0.011*	0.226
IL8	-0.087	0.050	0.081	0.398

IL6	-0.032	0.033	0.327	0.674
IL1ra	-0.029	0.049	0.563	0.721
IL1b	-0.073	0.039	0.062	0.398
HGF	-0.085	0.033	0.009*	0.226
IL9	0.012	0.048	0.800	0.863
IL7	-0.035	0.051	0.494	0.721
IL5	-0.103	0.051	0.045	0.398
IL4	-0.042	0.033	0.206	0.636
IL2ra	-0.090	0.049	0.067	0.398
IL2	-0.043	0.050	0.389	0.700
IFNg	-0.021	0.034	0.537	0.721
GROa	-0.078	0.095	0.410	0.700
GCSF	-0.010	0.034	0.768	0.851
FGFbasic	-0.032	0.033	0.329	0.674
Eotaxin	-0.023	0.033	0.490	0.721

SE, standard error. *P*.adjust. FDR corrected *P* value

The inverse variance weighted estimation method (IVW) was used in this direction, for seven or eight SNPs were available.

* *P* value of IL10 and HGF showed suggestive significance at the level of 0.05.
